# Perceptions of HIV-related health services in Zambia for people with disabilities who are HIV-positive

**DOI:** 10.7448/IAS.17.1.18806

**Published:** 2014-04-23

**Authors:** Stephanie A Nixon, Cathy Cameron, Jill Hanass-Hancock, Phillimon Simwaba, Patricia E Solomon, Virginia A Bond, Anitha Menon, Emma Richardson, Marianne Stevens, Elisse Zack

**Affiliations:** 1 International Centre for Disability and Rehabilitation, University of Toronto, Toronto, Canada; 2 Department of Physical Therapy, University of Toronto, Toronto, Canada; 3 Graduate Department of Rehabilitation Science, University of Toronto, Toronto, Canada; 4 Health Economics and HIV/AIDS Research Division (HEARD), University of KwaZulu-Natal, Durban, South Africa; 5 Dalla Lana School of Public Health, University of Toronto, Toronto, Canada; 6 Disability, HIV & AIDS Trust (DHAT), Harare, Zimbabwe; 7 School of Rehabilitation Science, McMaster University, Hamilton, Canada; 8 ZAMBART, Lusaka, Zambia; 9 Department of Global Health and Development, London School of Hygiene and Tropical Medicine, London, United Kingdom; 10 Department of Psychology, University of Zambia, Lusaka, Zambia; 11 Canadian Working Group on HIV and Rehabilitation, Toronto, Canada

**Keywords:** disability, stigma, vulnerability, blind, deaf, impairment, AIDS, equity

## Abstract

**Introduction:**

Despite the emerging body of literature on increased vulnerability to HIV among people with disabilities (PWDs), there is a dearth of evidence related to experiences of PWDs who have become HIV-positive. This priority was identified by a disability advocacy organization in Lusaka, Zambia, where the prevalence of HIV and of disability is each approximately 15%. The purpose of this study was to explore perceptions and experiences of HIV-related health services for PWDs who are also living with HIV in Lusaka, Zambia.

**Methods:**

This qualitative, interpretive study involved in-depth, semi-structured, one-on-one interviews with two groups of participants in Lusaka, Zambia: 21 PWDs who had become HIV-positive, and 11 people working in HIV and/or disability. PWDs had physical, hearing, visual and/or intellectual impairments. Interviews were conducted in English, Nyanja, Bemba or Zambian sign language. Descriptive and thematic analyses were conducted by a multidisciplinary, international research team.

**Results:**

Participants described their experiences with HIV-related health services in terms of the challenges they faced. In particular, they encountered three main challenges while seeking care and treatment: (1) disability-related discrimination heightened when seeking HIV services, (2) communication barriers and related concerns with confidentiality, and (3) movement and mobility challenges related to seeking care and collecting antiretroviral therapy. These experiences were further shaped by participants’ profound concerns about poverty and unmet basic needs.

**Discussion:**

This study demonstrates how PWDs who are HIV-positive have the same HIV care, treatment and support needs as able-bodied counterparts, but face avoidable barriers to care. Many challenges mirror concerns identified with HIV prevention, suggesting that efforts to promote inclusion and reduce stigma could have widespread benefits.

**Conclusions:**

Despite the growing body of literature on increased risk of exposure to HIV among HIV-negative PWDs, this is the first published study to examine perceptions of testing, treatment and other HIV services for PWDs who have become HIV-positive. Findings reveal far-reaching opportunities for improving the quality of care for this population.

## Introduction

Despite growing recognition of the HIV prevention needs of people with disabilities (PWDs), there is a dearth of evidence related to experiences of PWDs who have become HIV-positive. This is of particular concern in Zambia, where the prevalence of disability and of HIV are relatively high. This study focuses on health services for HIV-positive PWDs in Lusaka, Zambia, in order to identify strategies for mitigating barriers to access. We explored this issue by interviewing PWDs in Zambia who have become HIV-positive. We also interviewed service providers and policy makers working in the field of HIV and disability in Zambia to explore complementary perspectives on access to HIV-related health services for PWDs.

PWDs have historically been excluded from HIV planning and programming largely due to perceptions that they are not at risk. In 2004, however, the *Global Survey of HIV/AIDS and Disability Report* identified considerable risk of exposure to HIV among PWDs [1]. The report argued that PWDs are at least as at risk as able-bodied counterparts when considering factors known to exacerbate vulnerability to HIV, such as poverty, lack of education, sexual abuse, substance abuse, or precarious access to health care [1]. The report also contributed to debunking the widely held and inaccurate assumption that PWDs are not sexually active and, thus, not in need of sexual education. On the contrary, PWDs may be equally as sexually active as others and at increased risk of sexual abuse [2–10].

The relevance of HIV for PWDs was articulated in the *Disability and HIV Policy Brief* jointly released by UNAIDS, the World Health Organization and the Office of the High Commissioner for Human Rights in 2009 [11]. Since then, attention to HIV and disability has been on the rise [12–16]. However, official recognition of the links between HIV and disability has been only partially matched by research to understand implications for HIV service delivery and policy.

A growing body of research has explored aspects of HIV risk, vulnerability and prevention among PWDs [2–10,17] . Hanass-Hancock's systematic review of research on disability and HIV in Africa demonstrated an increase in publications between 2000 and 2008 [10]. Of the 36 studies reviewed, most focussed on prevention; no articles addressed interventions related to HIV treatment, care and support for PWDs. Much of the research described lower levels of HIV knowledge among PWDs, including misconceptions about transmission. The review also indicated low uptake of protective measures, such as condom use, and barriers in accessing HIV prevention services.

Despite the emerging body of literature on the prevention needs of PWDs who are HIV-negative, there is little evidence on the experiences of PWDs who have become HIV-positive. Information resides almost exclusively in reports produced by non-governmental organizations, disabled people's organizations and/or disability allies [18–23]. One exception is research on the prevalence of HIV among PWDs, which has been succinctly reviewed by Groce *et al*. [24]. They conclude that the data suggest at least an equal HIV prevalence rate for PWDs compared to non-disabled peers, although findings are too limited to draw definitive conclusions. They also note the lack of research on:

access to AIDS medication, care and support, issues which have already been flagged by disability researchers as areas in which people with disabilities may have been given lower priority. [24, p. 36]

The relationship between disability and HIV may be even more pronounced in Eastern and Southern Africa, where the countries with the highest HIV prevalence are also the countries with the highest disability prevalence [17]. However, with the exception of South Africa, little combined prevalence data are available for the region [17,24].

In Zambia, no official statistics exist on HIV prevalence among PWDs, which creates a challenge for understanding the extent of the pandemic in this population. However, the need for research on HIV-related health services among PWDs who live with HIV was identified as a priority by the Disability, HIV & AIDS Trust (DHAT), a disability advocacy organization in Lusaka [25,26]. Lusaka is a hyper-endemic setting with greater than 15% HIV prevalence among adults [27]. The prevalence of disability is difficult to determine due to limited and conflicting evidence, definitions of disability, and quality of data. The highest estimate of disability prevalence in Zambia – 14.5% – was made in the World Report on Disability, which used a broad definition of disability [28,29]. It is within this context of elevated rates of both HIV and disability that DHAT responded to calls for advocacy from its HIV-positive members related to HIV care and treatment for PWDs [20]. DHAT partnered with university-based researchers to undertake a programme of research and advocacy to better understand the health service needs of PWDs who are HIV-positive. The purpose of this study was to explore the perceptions of PWDs who are HIV-positive and of key informants (KIs) who work in HIV and disability regarding the provision of HIV-related health services for PWDs in Lusaka, Zambia. In particular, this analysis addresses the following question: what are the perceived challenges and, where possible, solutions related to accessing HIV-related health services for PWDs who are HIV-positive?

## Methods

This qualitative, interpretive study used in-depth, semi-structured, one-on-one interviews. We purposively sought two groups of participants for this study.

### Group 1: PWDs who are HIV-positive

Inclusion criteria for Group 1 were adults with disabilities in Lusaka, Zambia, who had subsequently become HIV-positive. We sought diversity in terms of gender and type of impairment (i.e., physical, hearing, visual and intellectual). For individuals with intellectual impairments, we included only participants who had the capacity to give informed consent and participate in conversations about their experiences. Recruitment was through posters and word-of-mouth within the HIV and the disability communities in Lusaka. We also used snowball sampling and asked existing participants to share study information with others who might meet inclusion criteria. Our Zambian research coordinator matched potential participants with one of six local, trained fieldworkers based on pragmatic (e.g., availability and location) and cultural (e.g., age and sex) considerations. Two of the fieldworkers were women with disabilities, two were HIV counsellors and two were certified Zambian sign language interpreters. All interviews with Group 1 participants were conducted in person and in English, Nyanja, Bemba or Zambian sign language according to the preference of the participant. Interviews with participants who were deaf or hard of hearing were conducted in Zambian sign language with the fieldworker simultaneously verbalizing the comments of both the interviewer and participant to audio-record the exchange. This process enhanced confidentiality by avoiding the need to engage a third person in the interview to interpret. Compensation was provided to participants.

### Group 2: KIs from the field of HIV and/or disability

Inclusion criteria for Group 2 were individuals (with or without disabilities) whose work in Lusaka was related to HIV and/or disability, including those working in community-based organizations or non-governmental organizations (e.g., AIDS service organizations or disabled people's organizations), HIV-related health services, or government positions with portfolios relevant to HIV and/or disability. These individuals were included in the study to elicit their perspectives regarding access to care for PWDs given their roles as service providers or policy makers. These participants were recruited purposively through the local expertise of our research team and collaborators. Potential participants were invited to participate in the study either by the Zambian Research Coordinator or the Principal Investigator (Nixon). All interviews were conducted in English by a member of the fieldwork team in person except for one interview that was conducted by the Principal Investigator by telephone. No compensation was provided for this group of participants because the Zambian members of the research team advised that this incentive was unnecessary.

All interviews were audiotaped. To facilitate collaborative data analysis among the international research team, we selected English as the common language in which all transcripts would be made available [30]. This was achieved through simultaneous transcription and translation of the eight interviews conducted in Nyanja and the four interviews conducted in Bemba. The transcriptionist listened to the digital audiofile in the source language and transcribed it directly into English. Any vernacular content that did not easily translate into English was maintained in the source language in brackets in the transcript. To increase cross-language trustworthiness [30], all transcripts were reviewed by both the Zambian research coordinator and the fieldworker who conducted the interview against the original audiofile to ensure accuracy and completeness of translation [31]. This study was approved by research ethics boards at the University of Zambia, University of Toronto in Canada and University of KwaZulu-Natal in South Africa.

### Data analysis

Data were analysed using a collaborative, multi-phase approach [32] to conventional content analysis [33]. A subset of the team (*n*=5) first developed, piloted and refined a coding framework based on concepts derived inductively from the Group 1 interviews following the DEPICT method described by Flicker and Nixon [34]. Each Group 1 transcript was then coded independently by two members of the research team [35] and inputted into a data organization software programme (NVivo 8.0©). Data from Group 2 were coded using a modified version of the coding framework relevant for the perspectives of these KIs working in the field. Descriptive summaries were developed that drew on both Group 1 and 2 findings related to specific coded categories (e.g., experiences in the health clinic queue, experiences with health workers). Finally, we iteratively reviewed the data relating to experiences with health services to inform the following analysis.

## Results

### Participants

Thirty-two participants were interviewed in Lusaka, Zambia, between August 2010 and June 2011 (see Table 1). Twenty-one participants were PWDs living with HIV (Group 1). Eleven participants were individuals working in fields related to HIV and/or disability, such as health services, community-based organizations, or government (Group 2). Several of the KIs in Group 2 had a disability, and thus were positioned to reflect on the interview questions both from their professional standpoint and also from personal experience. When presenting data below, quotes from Group 1 participants are identified as “PWD” followed by a participant number, and quotes from Group 2 participants are identified as “KI” followed by a participant number.

**Table 1:** Participant characteristics

**Group 1.**	**Number of participants**	**21 (66%)**
**People with disabilities in Lusaka,**	Sex	12 women (57%), 9 men (43%)
**Zambia, who are HIV-positive**	Age range	29–61 years old
***(PWD participants)***	*Type of impairment:*	
	Hearing	3 (14%)
	Mobility	12 (1 also had an intellectual impairment) (57%)
	Visual	4 (19%)
	Intellectual	2 (1 also had a mobility impairment) (9%)
	*Onset of disability:*	
	Acquired at birth or in childhood	15 (71%)
	Recently acquired	6 (29%)
**Group 2.**	**Number of participants**	**11 (34%)**
**People whose work in Lusaka, Zambia, is related to HIV and/or disability**	Sex	4 women (36%), 7 men (64%)
	*Type of organization:*	
*** (KI participants)***	HIV community organization	2 (18%)
	Disabled people's organization	5 (45%)
	Health service	2 (18%)
	Government department	2 (18%)
**Total sample**	**Number of participants**	**32**

### Perceptions regarding HIV-related services for PWDs

**Figure 1:**
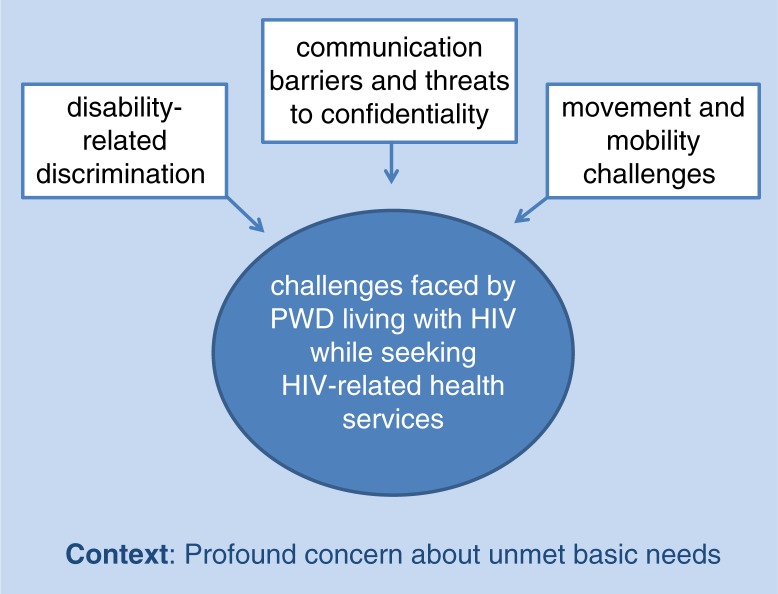
Challenges faced by PWDs living with HIV while seeking HIV-related health services.

Participants discussed their perceptions of health services across the HIV care continuum, including voluntary counselling and testing (VCT) during which they discovered they were HIV-positive, initiating and managing antiretroviral therapy (ART) over time, and other forms of HIV-related health support. They framed these experiences with HIV-related health services in terms of three types of challenges they encountered: disability-related discrimination, communication barriers, and movement challenges (see Figure 1). These experiences were further shaped by the context described by participants as profound concern about unmet basic needs. These findings are described below.

#### Disability-related discrimination while seeking HIV-related health services

Reports of stigma were pervasive in the data, with most PWD participants describing disability-related stigma that became heightened by the presence of HIV. There was a general perception that a person with a disability living with HIV would face “more rejection than a person who is able-bodied” (KI-19). Both PWDs and KI participants explained that witnessing PWDs seeking HIV services often sparked confusion and discomfort among able-bodied people. One participant noted:

Many people will be pointing a finger at an individual [with a disability] saying, ‘*Banatenga bwanji AIDS mwamene balili nabo aba?*’ [How could they contract AIDS considering their condition?] You know such things are being commented in our community. (KI-20)

Several KI participants explained that stigma arose from erroneous beliefs that PWDs are not at risk of exposure to HIV and, therefore, do not need access to HIV services. Assumptions appeared to be based, in part, on the idea that PWDs are not sexually active, resulting in the exclusion of PWDs (women and girls in particular) from HIV services. PWDs also reported that they were excluded from traditional rituals in which sex education is addressed. As one participant explained:

Some of this maybe is kind of cultural or social beliefs around disability and sexuality. I think a lot of people don't think that people with disabilities are sexual people. So, transmission can happen and sadly it's too late. So there's not even an awareness that everybody needs information. (KI-16)

The queues for HIV services were described by PWDs and KIs as a common location for experiencing acts of stigma. These two participants explained:

[Going to VCT] wasn't easy because a lot of people were staring at me just there on the queue. Others were passing comments. Honestly, I didn't find it easy. (PWD-11, female, physical impairment)

These drugs, we get them from health centres … but we are not all that free because we, we might even find the people who are waiting for the same drugs … you get some [stigmatizing] comments. We get discouraged in so many ways. (PWD-7, male, physical impairment)

Discrimination included taunting and name-calling linked to questioning why a person with a disability would need ART. One KI recounted a man with very short stature having insults hurled at him while he waited in the ART queue, including: “Why has this baby come on our day [instead of the day for paediatrics]?” (KI-20). In select cases, however, participants described PWDs receiving preferential care, such as being invited to jump the queue. In several cases, participants described stigma decreasing over time:

At the beginning, there were some difficulties as in seeing me as though I'm not their fellow human … now … we're just the same. (PWD-9, male, physical impairment)

One woman with a mobility impairment reported that her health worker was hesitant to provide her HIV-positive test results to her directly because of a presumed diminished capacity to understand the news:

They couldn't tell me straight that I was [diagnosed] HIV/AIDS. And I knew at last to say, maybe they don't want to tell me straight just because I am disabled. (PWD-10, female, physical impairment)

The desire for dignity and care “as a fellow human” was expressed widely in the data, including one participant who explained:

The first thing that I'd like is the emotional support … Yes. People should love me. They should care about me. (PWD-21, male, visual impairment)

Disability-related discrimination had dire consequences. Some participants described their experience in the queue as so upsetting that they considered discontinuing treatment. Others described the discrimination as so daunting that some PWDs chose not to seek VCT despite knowing that they could access ART if they tested HIV-positive. One participant explained:

They'll start, you know, victimizing me, talking about me, doing this and so instead, people just stay at home [and do not get tested] … In the end, OK you have to die with HIV/AIDS but a lot [of PWDs] are dying quietly, without even knowing whether what has killed them is HIV. (KI-26)

Participants identified several strategies for mitigating discrimination, including arranging for VCT services to be offered at disabled people's organizations where PWDs are known to convene. One disabled people's organization offered to provide “accessibility audits” to health clinics to identify barriers for PWDs. Participants were also aware of some HIV health workers requesting disability training.

#### Communication challenges as barriers to care and threats to confidentiality

Most participants described challenges related to communication for people with visual or hearing impairments seeking HIV-related health services. As this KI participant explained:

They should ensure that all people who deliver ARVs maintain confidentiality for blind people, who cannot read their manuals and cannot read what is written on the ARV packs. And then they should ensure that they maintain confidentiality for deaf people. In terms of IEC, Information and Education Communication, deaf people are still very very behind in as far as HIV/AIDS messages are concerned. Blind people do not get some of the messages which are shown as signs or signals on TV, that's another issue. (KI-27)

Participants who were deaf or hard of hearing described significant challenges in communicating with ART clinic staff in the absence of sign language translators.

Being disabled has affected my getting the services for HIV because there is an information breakdown, communication barriers. As a deaf [person], we don't access. I don't access any information. There is no one to interpret, so it means that it is not disability friendly. So those are not good services. I wish there was interpreters in these hospitals. (PWD-2, female, hearing impairment)

Several participants recounted stories of people being denied testing because health care workers felt unable to manage the encounter given communication constraints. One participant explained:

[We] received complaints from more than five deaf people where they've been turned back from, uh, VCT centres on the grounds that the counsellors were saying, ‘I cannot counsel you through writing because you'll be writing, I'll be writing, that will be slow’. And then if the deaf person says, ‘I cannot come with an interpreter because I need confidentiality’, the counsellors have refused’. (KI-27)

Another major concern among participants was the lack of confidentiality in the clinical encounter for people who were deaf or hard of hearing:

In counselling and even, you know, testing for HIV/AIDS, it's supposed to be private and voluntary. But for those of us that are deaf, you don't go alone. You're going with an [sign language] interpreter, and that interpreter is going to know about your, your [HIV] status. So it's not very private. (KI-26)

In response to this challenge, several participants described an innovative programme in Lusaka that simultaneously trained VCT counsellors in basic Zambian sign language, and trained Zambian sign language interpreters in VCT counselling in order to produce a cohort of workers able to provide VCT to people who are deaf without involving a third person in this sensitive exchange.

Several KIs recounted examples of confidentiality and privacy norms being broken by health care workers in the case of a person with a disability. As one explained:

Counsellors are not trained to handle disability and HIV/AIDS. They still have got that stigma that persons with disability should not go for counselling. So whenever they find a person with disability who is positive, it becomes a shock to them. They share it with other counsellors, and then it gets broken into society. (KI-27)

Barriers related to communication also included the inability to access written VCT consent or education materials for people who are blind. Participants explained how people with visual impairments were unable to read the label on ART medication and often feared asking for help from sighted peers due to risk of stigma. However, participants with visual impairments also described creative solutions using their sense of touch to identify their various prescription bottles.

Communication barriers were also raised by participants with physical impairments, including this PWD who said:

[Able-bodied people can access information during hospital visits but] compared to me and my fellow disabled persons, somehow to us it is somehow like hidden information. (PWD-14, male, physical impairment)

Only one PWD participant invoked the language of human rights in the context of his challenges to accessing ART:

One [issue] that I can say as a deaf [person] who has got the virus and then [compared to] someone who is able bodied, we have the same right even accessing medical support. It is my right as a deaf person to have a sign language interpreter in hospital for good communication, which means that we can be on the same level. As a deaf person, if I meet a medical staff it is very difficult for me to access information. That is, that is infringing my rights. So I … I really feel sign language interpreters must be trained who are proficient to interpret for the deaf even in medical centres. (PWD-1, male, hearing impairment)

#### Movement and mobility challenges

Various challenges related to movement and mobility were described by participants in the context of their experiences of seeking HIV-related health services. First, physical barriers were identified as impairing access to services for individuals with mobility impairments. Access barriers included steps leading into clinics or health series being offered on the second floor of a building with no elevator. As one participant explained:

Some of the places where the counselling and testing is done, it may be upstairs. Now, someone on a wheelchair won't, won't, it's either maybe you get out of the wheelchair, you start crawling up there, or maybe someone helps you. Or if there's no one to do that, then you just, you may reach the [health] centre, but you may not access the service that you are looking for because there's no proper, the environment is not conducive. (KI-26)

Participants also described how people with mobility or visual impairments often required assistance to travel to the clinic for care (e.g., for testing or to collect ART), which became onerous financially (e.g., paying double transport expenses for an assistant) or in terms of seeking favours (e.g. from neighbours). Various PWD participants described sending a friend or child to try to collect their ART, with varying degrees of success:

Especially on the part of [collecting] medicine, it's difficult for us, the disabled, because maybe the wheelchair gets damaged. When you send a child, they turn him down. Then two, maybe you're not feeling well, like I said, on food, you don't have strength for you to get up and go and collect medicine. (PWD-11, female, physical impairment)

Participants also described the requirements of collecting ART as physically onerous:

… I'd take maybe five hours just standing in the queue. They can't even get a chair … when our able-bodied friends reach there, they're quickly given the file and sometimes even a place to sit. (PWD-12, female, physical impairment)

One participant with a mobility impairment described on-going transport challenges resulting in repeatedly arriving late for clinic appointments, which led clinic staff to discontinue her treatment because she was viewed as an unsuitable ART candidate:

At the moment I have a problem because of transport. I do go to the clinic. I get on a bus. I live in [neighbourhood], I board from [location] going to [clinic]. So now when you are late, they'll tell you to come on another day. If it's today that they've told you to come on Saturday, you go on Saturday, they tell you that no, you are late, you're late. You should come on Monday. Again on Monday, if you are slightly late, again they'll tell you that you should come on Wednesday. Again on Wednesday if you go late, just like that. And if you go late five times, they tell you that you are not serious. Even when you explain to them that no, transport is difficult for me, for instance in the rainy season, meandering in the floods, it's difficult for me. But them, they don't understand. So me, they stopped giving me my file. (PWD-13, female, physical impairment)

Participants explained how they organized their lives to be able to mitigate challenges related to transport to seek HIV care. As one participant explained:

Being HIV positive and disability, there comes a time when you're very sick. Maybe it might, it comes into some sort of malaria. Now transportation becomes a very big problem, because the way I'm holding the crutches now, I'm feeling well. Once I'm sick, I cannot hold them. So that's why, that's where the family comes in, at least in our state. We have to be nearby our families. Because if you go to a place where no family lives, it becomes a burden to those neighbours once you're sick. Especially where transport is concerned, somebody will sacrifice to put you, to put you on his back, up to the right place. So it becomes a very, a very big problem. (PWD-7, male, physical impairment)

Mobility challenges were also raised by people with visual impairments:

I'm partially blind … I've got limited movement, so I cannot identify where you are … Maybe someone can escort me [to clinic] today, but the other time, that somebody cannot escort me because maybe somebody has got other things to do. (PWD-21, male, visual impairment)

#### Profound concern about unmet basic needs

The preceding results describe participants’ experiences of seeking HIV-related health care. Equally prevalent in the data, however, were participants’ accounts of the difficult context in which these experiences were located. That is, experiences of seeking health services for HIV care and treatment were described by participants as set against the backdrop of poverty and unmet basic needs. While experiences of poverty are not unique to PWDs in Zambia, these concerns were pervasive in the data.

The most pressing concern raised by participants was income insecurity and its impact on basic needs such as food, shelter and education. Very few examples were provided by either PWDs or KI participants of existing social support structures to assist with these essential needs. HIV home-based care was identified as a source of some support by two participants, and a third participant noted potential synergies between HIV home-based care and community-based rehabilitation, but explained that he had seen no integration of these services.

The need for food and nutrition was raised with frequency across both PWDs and KI participants, particularly in relation to taking ART. As one participant explained:

There are times when you don't have anything to eat at home. There, instead of taking [a meal] about three times a day, you'll take about two times a day. It depends, because after drinking it [ART] … if you haven't eaten anything, you'll even feel dizzy. What is required from these drugs, at least, is food. Food, at least if you know where to find food, no problem with those drugs. You cannot take them with your empty stomach, no. (PWD-7, male, physical impairment)

Another participant explained:

The change I've just experienced is whenever I take my medication, when I don't take enough food, I experience a lot of problems. Especially, myself I take the medicine in the evening, so during the day I have to go out with my duties and my work, but every evening, 19:00 hrs, I have to take my medicine. Now when I wake up in the morning, if I don't prepare anything on the table for me to eat, I find it very difficult to start a day … I come to hate the situation itself, because the medicine itself gives me a lot of problems of dizziness. I feel more weak in the morning, especially when I, I've not fed myself. So, those are the very serious problems I've been facing at the moment. (PWD-14, male, physical impairment)

This participant linked food insecurity with the challenge of collecting her ART:

You came having eaten only in the morning. The whole day you are just there [in line], maybe you don't even have money to buy food … and if you're on medication you need to eat frequently. (PWD-8, female, physical impairment)

Despite food insecurity, participants spontaneously discussed their commitment to adhering to ART. Motivations for adherence related to desire for continued health and, for women in particular, the need to stay alive to raise their children:

But me who is poor, even if I were to die today, who is going to take care of my children? It's better I just continue taking medication, I see the future and how I'm going to raise my children. (PWD-14, female, visual impairment)

## Discussion

This study demonstrates how PWDs may have the same HIV care, treatment and support needs as able-bodied counterparts, but face avoidable barriers in their efforts to access care. Physical and communication barriers appeared to be widespread. Even more daunting, however, were the reports of disability-related stigma, which were exacerbated through the process of seeking HIV services. Although 20 of 21 participants with disabilities were on ART, they also reflected that many of their peers experience such profound barriers that they are unable to access treatment, with dire consequences.

These findings build on and extend the literature in two important ways. First, research on HIV among PWDs has largely focussed on the *HIV prevention* needs of PWDs who are *HIV-negative* [2–10]. This study illuminates *HIV care, treatment and support* experiences of PWDs who are *HIV-positive*. The data emphasized physical, communication and attitudinal barriers to quality care, which have also been identified as barriers to HIV prevention and education [2,4,8,28,36–38], indicating that efforts to promote inclusion could have widespread benefits. Although most participants had access to ART, these challenges may influence their ability to seek care in the future, which has implications for treatment adherence.

Second, these challenges to quality HIV services are consistent with the longstanding body of evidence regarding lack of access to health care in general for PWDs. This is the case in resource-constrained settings [39] as well as countries with universal health care, like Canada [40]. A key aim of this article is to make visible the exclusion of PWDs from HIV-related health services to increase awareness of this problem. One necessary response is to better support health care workers in managing ART among PWDs as ART scale-up continues.

The case of Lusaka, Zambia, is also instructive. The Government of Zambia has adopted a number of progressive laws and policies pertaining to PWDs, including the Persons With Disabilities Act [41], a provision in the Zambian Constitution [42] and ratification of the Convention on the Rights of Persons With Disabilities in 2010 [43]. Zambia also recognized PWDs in the 2006–2010 National HIV/AIDS Strategy Framework [44]. As such, action on disability is required less in the form of official recognition and more in the practical implementation and enforcement of these commitments. As Zambia moves towards a treatment-as-prevention approach [45], and as the number of people living with HIV on treatment increases, addressing the needs of PWDs becomes even more pertinent. Countries without such progressive disability policies may have even further to go in delivering equitable HIV services.

### Implications for improving the quality of HIV-related services for PWDs

Clinicians, managers, policy makers and funders involved in the delivery of HIV-related services can facilitate access within the clinic setting in three key ways: reducing stigmatizing attitudes related to disability; addressing physical mobility and transport barriers to care; and recognizing communication challenges, especially for people with visual or hearing impairments [46].

Historic lessons from disability activism can inform this contemporary concern. Like the Greater Involvement of People Living with HIV (GIPA) principle [47], the greater inclusion of PWDs [48] and their allies in responding to access barriers will result in more relevant solutions and the reduction of stigma through participation. Disabled people's organizations represent a resource whose expertise is underrepresented in the HIV response [49].

Furthermore, the vicious cycle of poverty, food insecurity and HIV [50,51] is exacerbated by the addition of disability [52]. In particular, this study flags the significance of food security in supporting ART adherence among PWDs.

Finally, an additional strategy for ensuring that the needs and rights of PWDs are addressed by HIV services is to ensure that PWDs are recognized in National HIV Strategic Plans, as exemplified by the Government of Zambia [53]. The “Framework for the Inclusion of Disability in the National Strategic Plans on HIV and AIDS” is a comprehensive tool for ensuring disability-inclusive plans [15]. Once plans are in place, however, accountability mechanisms are required to ensure that these policies are applied in practice [54].

### Directions for future research

There is a pressing need for research that explores interventions and processes for reducing disability-related discrimination, which may draw lessons from successes within the field of HIV stigma reduction. Second, research should examine relevant and sustainable responses to the basic food and shelter needs of PWDs who are HIV-positive with particular attention to gendered experiences of poverty. Third, while we intentionally sought a range of impairment types in our sample, future research should explore in detail the unique issues facing people with similar impairments. Finally, longitudinal research is needed to map the experiences of disablement among able-bodied people on ART over time in resource-poor settings like Zambia. Given the potential for people living longer with HIV to experience disability as a result of HIV, their needs may become aligned with people with pre-existing disabilities who have become HIV-positive.

### Limitations

All but one of the HIV-positive PWDs who participated in this study were on ART. We do not expect that this reflects coverage of ART among PWDs in Zambia since PWDs on ART may have been more likely than others to participate in this study. As such, we caution against interpreting findings as reflecting the experiences of HIV-positive PWDs who are not on ART. Furthermore, the barriers in accessing HIV-related services may be amplified for HIV-positive PWDs who were not recruited for this study. A second limitation is that we are unable to contrast our findings with able-bodied people in the same area. This is particularly salient regarding the findings related to food security. We also note that several of the KIs were also PWDs and/or affiliated with disabled people's organizations, which may have led these individuals to include personal experiences related to their own exclusion in addition to reflections about HIV-positive PWDs with whom they work.

## Conclusions

This is the first study to examine the HIV-related health service experiences of women and men with disabilities who are HIV-positive. Despite the growing body of literature focussed on HIV vulnerability, risk and prevention among HIV-negative PWDs, there has yet to be an empirical study examining VCT, ART and other HIV care services focussed on the needs of PWDs who have become HIV-positive. Findings reveal tremendous and feasible opportunities for improving the quality of care for this population.
